# Ultraviolet radiation exposure time and intensity modulate tomato resistance to herbivory through activation of jasmonic acid signaling

**DOI:** 10.1093/jxb/ery347

**Published:** 2018-10-09

**Authors:** Rocío Escobar-Bravo, Gang Chen, Hye Kyong Kim, Katharina Grosser, Nicole M van Dam, Kirsten A Leiss, Peter G L Klinkhamer

**Affiliations:** 1 Plant Science and Natural Products, Institute of Biology Leiden (IBL), Leiden University, Leiden, The Netherlands; 2 Molecular Interaction Ecology, German Center for Integrative Biodiversity Research (iDiv), Leipzig, Germany; 3 Friedrich Schiller University Jena, Institute of Biodiversity, Jena, Germany

**Keywords:** Glandular trichomes, herbivores, jasmonic acid, phenolics, plant defenses, salicylic acid, thrips, tomato, Western flower thrips

## Abstract

Ultraviolet (UV) radiation can modulate plant defenses against herbivorous arthropods. We investigated how different UV exposure times and irradiance intensities affected tomato (*Solanum lycopersicum*) resistance to thrips (*Frankliniella occidentalis*) by assessing UV effects on thrips-associated damage and host-selection, selected metabolite and phytohormone contents, expression of defense-related genes, and trichome density and chemistry, the latter having dual roles in defense and UV protection. Short UV daily exposure times increased thrips resistance in the cultivar ‘Moneymaker’ but this could not be explained by changes in the contents of selected leaf polyphenols or terpenes, nor by trichome-associated defenses. UV irradiance intensity also affected resistance to thrips. Further analyses using the tomato mutants *def-1*, impaired in jasmonic acid (JA) biosynthesis, *od-2*, defective in the production of functional type-VI trichomes, and their wild-type, ‘Castlemart’, showed that UV enhanced thrips resistance in Moneymaker and *od-2,* but not in *def-1* and Castlemart. UV increased salicylic acid (SA) and JA-isoleucine concentrations, and increased expression of SA- and JA-associated genes in Moneymaker, while inducing expression of JA-defensive genes in *od-2*. Our results demonstrate that UV-mediated enhancement of tomato resistance to thrips is probably associated with the activation of JA-associated signaling, but not with plant secondary metabolism or trichome-related traits.

## Teaser

Supplemental ultraviolet radiation increases tomato resistance to thrips herbivory through activation of jasmonic acid signaling, and without changes in the plant metabolome or the density of type-VI trichomes and their associated volatiles.

## Introduction

Ultraviolet (UV) radiation (280–400 nm), a small fraction of the solar radiation reaching the terrestrial ecosystems, is an important modulator of plant physiology (Paul and Gwynn-Jones, 2003). In particular, UV-B (280–315 nm) can decrease leaf size and stem elongation, increase axillary branching and leaf thickness, and induce UV-absorbing compounds such as flavonoids and phenolic acids (Jenkins, 2009; Robson *et al.*, 2015). Leaf epidermal accumulation of phenolics reduces both oxidative damage and UV-B penetration to the inner photosynthetic layers (Agati and Tattini, 2010), protecting the photosynthetic apparatus from damaging doses. These photomorphogenic responses are mediated by the activation of the UV-B photoreceptor UV RESISTANCE LOCUS 8 (UVR8) (Rizzini *et al.*, 2011) upon plant exposure to low or moderate UV-B (Hectors *et al.*, 2007; Brown and Jenkins, 2008).

Acclimation responses to enriched UV-B conditions can affect plant performance and interactions with herbivores. UV-B exclusion and supplemental studies have shown that it can decrease preference and/or performance of herbivorous arthropods in diverse plant species (Mazza *et al.*, 1999; Rousseaux *et al.*, 2004; Caputo *et al.*, 2006; Foggo *et al.*, 2007; Demkura *et al.*, 2010; Kuhlmann and Müller, 2010; Mewis *et al.*, 2012; Mazza *et al.*, 2013; Zavala *et al.*, 2015). This has been partially explained by UV-B-mediated effects on constitutive plant defenses, such as increases in secondary metabolites (Escobar-Bravo *et al.*, 2017*b*). In addition, UV-B has been reported to increase the density of leaf trichomes (Barnes *et al.*, 1996; Yan *et al.*, 2012), which are hairy epidermal structures involved in plant resistance to herbivory and in protection against abiotic stresses such as excess light intensity and UV (Karabourniotis *et al.*, 1993, Glas *et al.*, 2012). However, whether UV-mediated increases in trichome density enhances plant resistance to herbivores has not yet been tested.

Plant inducible defense responses to herbivory are also affected by UV (Ballaré, 2014). These responses are mediated by the activation of the jasmonic acid (JA), salicylic acid (SA), ethylene (ET), and abscisic acid (ABA) signaling pathways, and are fine-tuned by these or other phytohormones such as auxins, cytokinins, and gibberellins (Pieterse *et al.*, 2012). Activation of the JA signaling pathway is associated with increased plant resistance to leaf-chewing, piercing–sucking, and some phloem-feeding arthropods (Howe and Jander, 2008). UV-B can increase constitutive (i.e. pre-herbivory) and also inducible (i.e. post-herbivory) jasmonate levels, resulting in reinforced chemical defenses (Đinh *et al*., 2013; Qi *et al.*, 2018). Recently, Dillon *et al.* (2018) reported enhanced induction of ET by combined solar UV-B and herbivory, and highlighted its possible role in soybean defenses. Less is known about the roles of SA, auxin, and ABA in UV-mediated induction of plant defenses. However, auxin and ABA are regulatory components of the UV-mediated accumulation of flavonoids in Arabidopsis and grape (*Vitis vinifera*), respectively (Berli *et al.*, 2010; Hectors *et al.*, 2012), and SA levels are induced with increased UV irradiation times (Mackerness *et al*., 1999).

Under natural conditions, plants are simultaneously exposed to UV-B and UV-A (315–400 nm), both of which probably affect plant defense responses. UV-A radiation has different effects on plant physiology when compared to the more-energetic UV-B (Morales *et al.*, 2010, 2011, 2013; Escobar-Bravo *et al.*, 2017*b*). Furthermore, UV-A can attenuate UV-B-associated deleterious effects on the photosynthetic apparatus under conditions of low photosynthetically active radiation (PAR) (Adamse *et al.*, 1994; Joshi *et al.*, 2007; Štroch *et al.*, 2015) or, combined with UV-B, it can increase plant photosynthetic performance (Tsormpatsidis *et al.*, 2008).

Despite our increasing knowledge of the effects of UV-B on plant defenses against herbivorous arthropods, how different doses (A+B) modulate plant defenses against herbivory is unknown. Plant responses depend on the UV acclimation state, exposure time (long- versus short-term), radiation intensity, the PAR:UV ratio, and the plant species (de la Rosa *et al.*, 2001; Frohnmeyer and Staiger, 2003; Brown and Jenkins, 2008). High UV-B exposure results in gene expression profiles that are markedly different from those observed in plants acclimated to low doses (Ulm *et al.*, 2004; Hectors *et al.*, 2007). High UV-B fluence rates can induce the production of reactive oxygen species (ROS) and cause damage to DNA, proteins, membranes, and lipids (Brown and Jenkins, 2008; Hideg *et al.*, 2013), resulting in harmful effects on plant performance. Furthermore, while solar UV positively influences plant resistance to herbivores, this might come at the expense of reduced growth by antagonizing auxin signaling (Hectors *et al.*, 2012; Hayes *et al.*, 2014). These UV-mediated plant responses need to be disentangled when aiming for the efficient use of UV in crop production.

Here, we used cultivated tomato (*Solanum lycopersicum*) to study the effects of different UV doses (i.e. daily exposure times and irradiance intensities) and a fixed UV B:A ratio (~1:1.2) on plant resistance to Western flower thrips (*Frankliniella occidentalis*), an important insect pest of many vegetable and ornamental crops worldwide (Steenbergen *et al.*, 2018). Despite being a major crop species, the effects of UV radiation on tomato physiology and resistance to relevant biological stresses have not been investigated. Furthermore, it is not known whether UV might affect the glandular trichomes on tomato leaves, which represent an important chemical and physical barrier against herbivory (Kang *et al.*, 2010*a*, 2010*b*). The availability of mutants deficient in JA biosynthesis or glandular trichomes makes cultivated tomato an ideal system to assess the effects of UV on plant resistance to herbivorous arthropods. We first tested the effects of different supplemental UV daily exposure times on resistance to thrips and selected the most effective dose to study the dynamics of the induced resistance. Time-course experiments were used to assess UV effects on thrips-associated damage and preference, on specific UV-absorbing compounds, on the metabolome, and on defense-related type-VI leaf glandular trichomes. We then studied the effects of two UV irradiance intensities, but with the same daily exposure times, on tomato growth and resistance, and examined the mechanism of this induction by using mutants defective in JA signaling (*defenseless-1*) and trichome-associate defenses (*odorless-2*). Further analyses of auxin, ABA, SA, and jasmonates levels, as well as the expression of defense-responsive genes, were used to determine the direct effects of UV on plant growth- and defense-related signaling pathways. Our study has significant implications for agricultural systems, as the use of artificial lighting to optimize crop yield is currently increasing in areas where light intensity is significantly reduced during the winter months (e.g. boreal and temperate climatic zones) and in greenhouses depleted of UV due to the use of non-transmitting UV materials (Vänninen *et al.*, 2010; Wargent and Jordan, 2013). Determining how plants respond to sudden UV exposure is then crucial in the context of new lighting technologies and greenhouse-grown crops such as tomato (Mariz-Ponte *et al.*, 2018).

## Materials and methods

### Plant and insect materials

Experiments were performed using the following tomato (*Solanum lycopersicum*) genotypes: cv. ‘Moneymaker’, and the jasmonate-deficient mutant *defenseless-1* (*def-1*) (Lightner *et al.*, 1993; Howe *et al.*, 1996) and the trichome-deficient mutant *odorless-2* (*od-2*) (Kang *et al.*, 2010*a*) (both kindly provided by Prof. Gregg Howe, Michigan State University), as well as their wild-type, cv. ‘Castlemart’. Seeds were sown in plastic trays filled with potting soil (Horticoop, Lentse Potgrond, The Netherlands) and placed in a climate room with 113.6 µmol m^–2^ s^−1^ of PAR and a photoperiod of 16/8 h light/dark, at 20 °C and 70% RH. Plantlets were transplanted into individual plastic pots (11 × 11 × 12 cm) containing the same potting soil at 15 d after germination.

Western flower thrips (*Frankliniella occidentalis*) [Pergande] were obtained from a colony reared on chrysanthemum flowers maintained in a climate room at 25 °C, 70% RH, and a photoperiod of 16/8 h.

### UV daily dose exposure experiments

To test the effects of different daily UV exposures on tomato resistance to thrips two independent experiments were performed. In both experiments, 4-week-old Moneymaker plants at the 3–4 leaf-stage were randomly transferred to two climate cabinets (Snyders Scientific B.V.) provided with 113.6 ± 10 µmol m^–2^ s^−1^ of PAR, a photoperiod of 16/8 h, 20 °C, and 70% RH. In one of the cabinets, UV radiation was supplied by two fluorescent lamps (Philips broadband TL 40/12RS) suspended at 65 cm above the plants. The lamps were wrapped with white cellulose paper filters (40 g m^–2^; Rachow Kunststoff-Folien, Hamburg, Germany) that attenuated the UV intensity and excluded damaging UV-C radiation (λ<280 nm) (Supplementary Fig. S1 at *JXB* online). In the first experiment, plants (*n*=5) were subjected to the following UV regimes for a period of 14 d: (1) 0 kJ m^−2^ d^−1^, (2) 0.34 kJ m^−2^ d^−1^, (3) 0.69 kJ m^−2^ d^−1^, (4) 1.36 kJ m^−2^ d^−1^, or (5) 2.04 kJ m^−2^ d^−1^. This was achieved by exposure to 0, 30, 60, 120, or 180 min d^−1^ of UV, respectively. In the second experiment (*n*=6–7), the following UV regimes were applied for 14 d: (1) 0 kJ m^−2^ d^−1^, (2) 0.17 kJ m^−2^ d^−1^, (3) 0.34 kJ m^−2^ d^−1^, or (4) 0.51 kJ m^−2^ d^−1^. For this, plants were irradiated for 0, 15, 30, or 45 min d^−1^, respectively. Application of UV started midway through the 16-h light period. In both experiments, control- and UV-treated plants were subjected to non-choice whole-plant bioassays of thrips damage at 14 d after the beginning of the treatment (see below).

The UV spectral irradiance (280–400 nm) was measured and integrated using a spectroradiometer (Flame-S, Ocean Optics). As the position of the UV lamps was not changed, the irradiance intensity received by the plants increased as they grew towards the light source. UV measurements were therefore performed at 0, 3, 7, 10, and 14 d at the plant canopy level (Supplementary Table S1) and were used to calculate the mean intensity received by the plants during the experiments. To minimize the damaging effects of high UV-B doses, both the PAR and daily UV levels were set to be as much as 10 times lower than those observed in natural field conditions (i.e. 1000–1500 µmol m^–2^ s^−1^ and 5.5 kJ m^−2^ d^−1^, respectively), and the UV-B irradiance was 40 times lower than that usually observed in the field at noon (i.e. 300–400 μW cm^−2^) (Demkura *et al.*, 2010; Đinh *et al*., 2013; Barnes *et al.*, 2016).

### UV time-course experiments

Moneymaker plants at 4 weeks old (3–4 leaf-stage) were randomly transferred to control or UV regimes as described above. In the UV regime, plants were exposed to 30 min d^−1^ of UV for 14 d. This treatment was selected because it was found to have the strongest positive effect on plant resistance to thrips in the UV dose experiments. At 1, 3, 7, 10, and 14 d after the beginning of the treatment, control and treated plants were subjected to non-choice whole-plant thrips bioassays (*n*=5). Another group of control and treated plants were used for dual-choice leaf-disc bioassays, or sampled for LC/MS analysis of rutin and chlorogenic acid levels, non-targeted metabolic NMR analysis, and determination of type-VI trichome density and their associated volatiles by GC/MS.

### UV irradiance intensity–dose experiments

We further determined whether variations in the UV irradiance intensity experienced by the plants in the time-course experiments due to their increased proximity to the UV source during their growth affected the defenses against thrips. For this, plant responses to two UV irradiance intensities corresponding to the light regimes received by plants at day 1 (0.30 kJ m^−2^ d^−1^) and day 14 (0.422 kJ m^−2^ d^−1^) in the time-course experiment (Supplementary Table S1) were tested using the same UV exposure time per day (30 min). In addition, we investigated whether these UV regimes, hereafter referred to as low- and high-UV, could enhance defenses against thrips in Moneymaker, and in the herbivore-susceptible genotypes *def-1* and *od-2*, as well as their wild-type ‘Castlemart’. The UV regimes were supplied by two fluorescent lamps (Philips broadband TL 40/12RS) in a climate cabinet (Snyders Scientific B.V.) provided with 113.6 ± 10 µmol m^–2^ s^−1^ of PAR, a photoperiod of 16/8 h, 20 °C, and 70% RH. The UV cabinet was divided in two separated areas where low- or high-UV were applied by adjusting the distance between the plants and the UV lamps. The PAR levels were adjusted to be similar in the control (no UV), low-, and high-UV conditions. Seeds were sown in plastic trays and placed under control, low-, or high-UV conditions for a total period of 28 d. At 2 weeks after the beginning of the treatments, seedlings were transplanted to plastic pots (9 × 9 × 10 cm) containing potting soil as described above. At 28 d after the beginning of the treatments, plants were used for non-choice whole-plant thrips bioassays, determination of plant growth parameters (number of leaves and stem length above the cotyledons), type-VI trichome densities, hormone contents, and gene expression analyses.

### Non-choice whole-plant bioassays of thrips damage

Plants were individually placed into thrips-proof cages as described by Leiss *et al.* (2009). All cages were randomly placed in a climate room provided with 113.6 ± 10 µmol m^−2^ s^−1^ of PAR, a photoperiod of 16/8 h, 25 °C, and 70% RH. Each plant was infested with 20 thrips (18 females and two males) as described by Escobar-Bravo *et al.* (2017*a*). After 7 d, thrips feeding damage to the leaves (‘silver damage’) was evaluated for the whole plant and expressed as mm^2^ per plant.

### Dual-choice leaf-disc bioassays of thrips preference

Dual-choice leaf-disc assays (Escobar-Bravo *et al.*, 2017*a*) were used to test thrips preference for samples taken from the third/fourth youngest leaf of control or UV-treated Moneymaker plants at 1, 3, 5, 7, 10, and 14 d after the beginning of the treatment in the UV time-course experiment (*n*=5).

### LC/MS analysis of leaf contents of rutin and chlorogenic acid

We examined changes in levels of two of the major phenolic compounds of tomato leaves, rutin and chlorogenic acid, (Larbat *et al.*, 2014) in control and UV-treated Moneymaker plants at 1, 3, 7, 10, and 14 d after the beginning of the treatment in the UV time-course experiment. This chemical analysis was replicated in a second time-course experiment (*n*=6–10). Analysis was performed on leaflets taken from the third/fourth youngest leaf from the apex. Samples were flash-frozen in liquid nitrogen and stored at –80 °C until analysis. Before extraction, samples were freeze-dried, homogenized, and aliquoted into 2-ml Eppendorf tubes (10 mg plant material per tube). The samples were extracted with 1.5 ml of 80% methanol aqueous solution, vortexed for 45 s, sonicated for 10 min, and centrifuged at 18 890 *g* for 20 min at 4 °C. The supernatant was filtered through a 45-mm syringe filter and 5 μl was used for LC/MS analysis, which was performed using a micrOTOF-QII (Bruker Daltonics GmbH, Germany) coupled to an Ultimate 3000 RS (ThermoScientific, USA) UHPLC quaternary pump with a diode array detector. Detection was carried out using electrospray ionization in negative mode over the mass range of *m*/*z* 120–1200. Reverse-phase liquid chromatographic separation was performed using a Kinetex C18 column (100 × 2.10 mm, 2.6-μm particles) (Phenomenex, USA) maintained at 30 °C. An elution gradient consisting of 0.1% formic acid (Optima, Fisher Scientific) in MilliQ water (solvent A) and methanol (Merck Millipore, Germany) +0.1% formic acid (solvent B) was used. The gradient profile used an initial condition of 10% B, 12 min linear gradient to 65% B, 4 min ramp to 90% B, 1 min hold at 90% B, and return to 10% B over 1.1 min, resulting in a run time of 18.1 min per sample. The flow rate was 0.35 ml min^−1^. Quantification was performed at 254 nm for rutin and at 326 nm for chlorogenic acid using calibration curves for both compounds (Sigma-Aldrich).

### NMR analysis

NMR analysis was performed on leaflets taken from the third/fourth youngest leaf from the apex in control and UV-treated Moneymaker plants (*n*=5) at 14 d after the beginning of the treatment in the UV time-course experiment. Samples of 10 mg of freeze-dried plant material were extracted with 1 ml of KH_2_PO_4_ buffer in D_2_O (pH 6) containing 0.05% trimethylsilane propionic acid sodium salt and CH_3_OH-*d*_4_ (1:1). Extracts were vortexed, sonicated for 20 min, and centrifuged at 18 890 *g* for 10 min at room temperature. Then 300 μl of the supernatant was transferred to NMR-tubes for spectral analysis. ^1^H NMR spectra were recorded at 25 °C on a 600 MHz Bruker AV 600 spectrometer equipped with cryo-probe operating at a proton NMR frequency of 600 MHz, and analysed as described by López-Gresa *et al.* (2012).

### Determination of type-VI trichome density

We tested whether type-VI glandular trichomes were affected in the UV time-course and irradiance intensity experiments. Trichome density was measured on the adaxial and/or abaxial surfaces of leaflets taken from the third/fourth youngest, fully expanded leaf (*n*=5) following the methodology described by Escobar-Bravo *et al.* (2017*a*).

### GC/MS analysis of terpene content in trichome-derived exudates

Terpene production by type-VI glandular trichomes was analysed in leaf exudates collected from two leaflets belonging to the same leaf that was used for trichome density measurements in the UV time-course experiment (*n*=5). Leaf fresh weight was measured before extraction. Trichome-derived exudates were obtained by placing leaves in 2 ml pentane containing 10 μg of benzyl acetate as an internal standard (Schilmiller *et al.*, 2009; Kang *et al.*, 2010*a*, 2010*b*; Sallaud *et al.*, 2012) and gently shaking for 2 min before removal. A 1-μl sample from the resulting extract was injected into an Agilent model 7890 gas chromatograph fitted with a 5975C inert XL MSD Triple Axis Detector using a split ratio of 20:1. The injector temperature was 280 °C. The initial temperature of the column (30 m × 0.25 mm, 0.25 μm film thickness; DB-5MS, Agilent Technologies) was set at 40 °C, then ramped to 150 °C at 15 °C min^–1^ and finally to 280 °C at 3 °C min^–1^. The helium carrier gas flow was 1.6 ml min^–1^. Terpenes were identified by comparison with authentic standards or by comparison with retention times and spectral information available in the Agilent GC/MSD ChemStation software. Compounds were quantified on the basis of the internal standards. Calibration curves of known concentrations (five data points in the range of 0.5–60 μg) of synthetic external standards were generated to calculate the internal response factor (IRF). For this, α-pinene and β-caryophyllene (Sigma-Aldrich) were used as external standards to determine the IRF of monoterpenes and sesquiterpenes, respectively. Terpene concentrations were calculated as: Amount of specific compound = (Amount IS × Area SC × IRF)/Area IS, where IS is internal standard and SC is the specific compound of interest. Terpene contents was expressed on the basis of fresh leaf weight.

### Hormone analysis

To investigate the signaling pathways involved in the UV-mediated induction of defenses against thrips, we determined how high-UV irradiance intensity (which strongly reduced silver damage in Moneymaker and *od-2*) influenced plant defense- and growth-related hormones levels at 28 d after the beginning of the treatment. The concentrations of 12-oxo-phytodienoic acid (OPDA), JA, jasmonic acid-isoleucine (JA-Ile), SA, ABA, and auxin (indole-3-acetic acid, IAA) were analysed in leaflets taken from the third/fourth youngest leaf of control and high UV-treated plants (*n*=5) following the procedures described by Machado *et al.* (2013) and Schäfer *et al.* (2016) with some modifications. Approximately 100 mg of frozen and homogenized leaf material was aliquoted in 2-ml Eppendorf tubes and extracted with 1 ml of ethyl acetate containing 40 ng of the phytohormone standards D_6_-ABA (Olchemin), D_6_-JA (HPC), D_6_-JA-Ile (HPC), D_6_-SA (Olchemin), and D_5_-IAA (Olchemin). Samples were vortexed for 10 min and centrifuged at 18 994 *g* for 10 min at 4 °C. The supernatants were transferred to new Eppendorf tubes and evaporated to dryness in a vacuum-concentrator at room temperature. The residue was dissolved in 0.2 ml of 70% methanol (v/v) for 5 min using an ultrasonic bath, and centrifugated 5 min at 18994 *g*. The supernatants were transferred to glass vials and then analysed by LC-MS/MS (EVOQ, Bruker), with 20 µl of each sample being injected onto a C18 Zorbax column (4.6 × 50 mm, 1.8 µm, 600 bar). The mobile phase was comprised of solvent A [0.05 % (v/v) formic acid in LCMS-grade water] and solvent B [0.05% (v/v) formic acid in LCMS-grade methanol]. The program had a constant flow rate of 0.4 ml min^–1^ and consisted of 0–0.5 min 95% solvent A; 0.5–2.5 min 50% solvent A, and 50% solvent B; 2.5–3.5 100% solvent B; 3.5–4.5 min 95% solvent A. The column temperature was set at 42 °C. The cone, probe, and nebulizer gas were set at the following flow conditions (arbitrary units/temperature): 35/350 °C, 60/475 °C, and 60 (arbitrary unit), respectively. Phytohormones were measured by monitoring the transition *m*/*z* described in Supplementary Table S2 and were quantified using the signal of their corresponding internal standard, and expressed on the basis of fresh leaf weight.

### Gene expression analysis

Expression of the JA-responsive genes *Threonine deaminase* (*TD-2*) and *jasmonate inducible protein-21* (*JIP-21*) were analysed in control and high UV-treated plants at 28 d after the beginning of the treatment. Because SA levels were induced in high UV-treated plants, expression of the SA-associated marker *pathogenesis-related protein-6* (*PR-P6*) was also analysed (Alba *et al.*, 2015). Total RNA was isolated following the protocol described by Verwoerd *et al.* (1989). DNAse treatment, cDNA synthesis, and qRT-PCRs were performed following the procedures described by Escobar-Bravo *et al.* (2017*a*). Five biological replicates with two technical replicates were analysed per treatment. *Actin* was used as a reference gene. The normalized expression (NE) was calculated using the Δ*C*_t_ method described by Alba *et al.* (2015). NE values were scaled to the lowest average NE, which was set to 1. Gene-specific primers are shown in Supplementary Table S3.

### Statistical analysis

Statistical analyses were performed using SPSS software (version 21; SPSS Inc., Chicago, IL, USA). Data residuals were first analysed using Levene and Kolmogorov–Smirnov tests to determine the heteroscedasticity of variance and normality, respectively. The effects of daily UV exposure times on silver damage were tested using generalized linear models (GLMs), using linear distribution and identity link functions, followed by Fisher’s least-significant difference (LSD) *post hoc* test. For the time-course experiment, differences in silver damage, type-VI trichome densities, trichome-derived terpenes, and leaf contents of rutin and chlorogenic acid between control and UV-treated plants at each time point were tested using Student’s *t*-test. Thrips preference was tested by paired *t*-tests. Patterns of chemical signals detected by NMR in leaf extracts of control and UV-treated plants were subjected to principal component analysis (PCA) using SIMCA-P 13 software (Umetrics, Sweden). Variables were centered and scaled to unit variance. For the irradiance intensity experiments, the effects of plant genotype, UV intensity, and their interaction on silver damage, leaf number, stem length, type-VI trichome density, hormone content, and NE were tested by GLM, using linear distribution and identity link functions, followed by LSD *post hoc* tests. Data for NE of *TD-2* and *JIP-21* were log- and log(*x*+0.00001)-transformed before analysis, respectively.

## Results

### UV-mediated enhancement of plant resistance to thrips depends on the duration of daily exposure

**Fig. 1.**
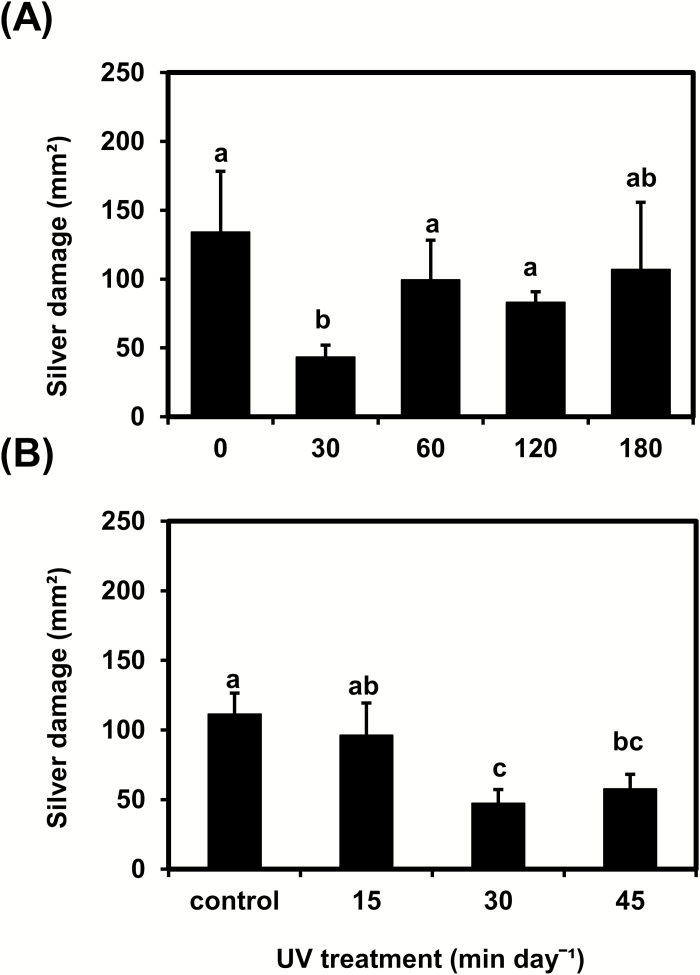
Thrips-associated damage (‘silver damage’ symptoms) in tomato ‘Moneymaker’ plants irradiated with supplemental UV for (A) 0, 30, 60, 120, or 180 min d^−1^, or (B) 0, 15, 30, or 45 min d^−1^ for a period of 14 d and subsequently used for non-choice whole-plant thrips bioassays. Silver damage was evaluated at 7 d after thrips infestation. Data are means (+SEM) of *n* = 4–5 individual plants in (A) and *n* = 6–7 individual plants in (B). Different letters indicate significant differences among groups as determined by GLM followed by Fisher’s LSD test (*P*≤0.05).

Variations in daily UV exposure time significantly affected tomato resistance to thrips (Fig. 1). Plants irradiated with UV for 30 min (0.34 kJ m^−2^ d^−1^) over 14 consecutive days showed less silver damage symptoms than control plants or plants irradiated for longer daily periods (60, 120, or 180 min; *P*<0.05) (Fig. 1A). To more precisely identify the most effective UV dose for increasing resistance, a second experiment testing the effect of different daily doses (0, 15, 30, and 45 min) was carried out. Again, plants irradiated for 30 min showed the lowest silver damage symptoms (*P*<0.05) (Fig. 1B).

### UV-mediated enhancement of plant resistance and repellency against thrips increases with time

**Fig. 2.**
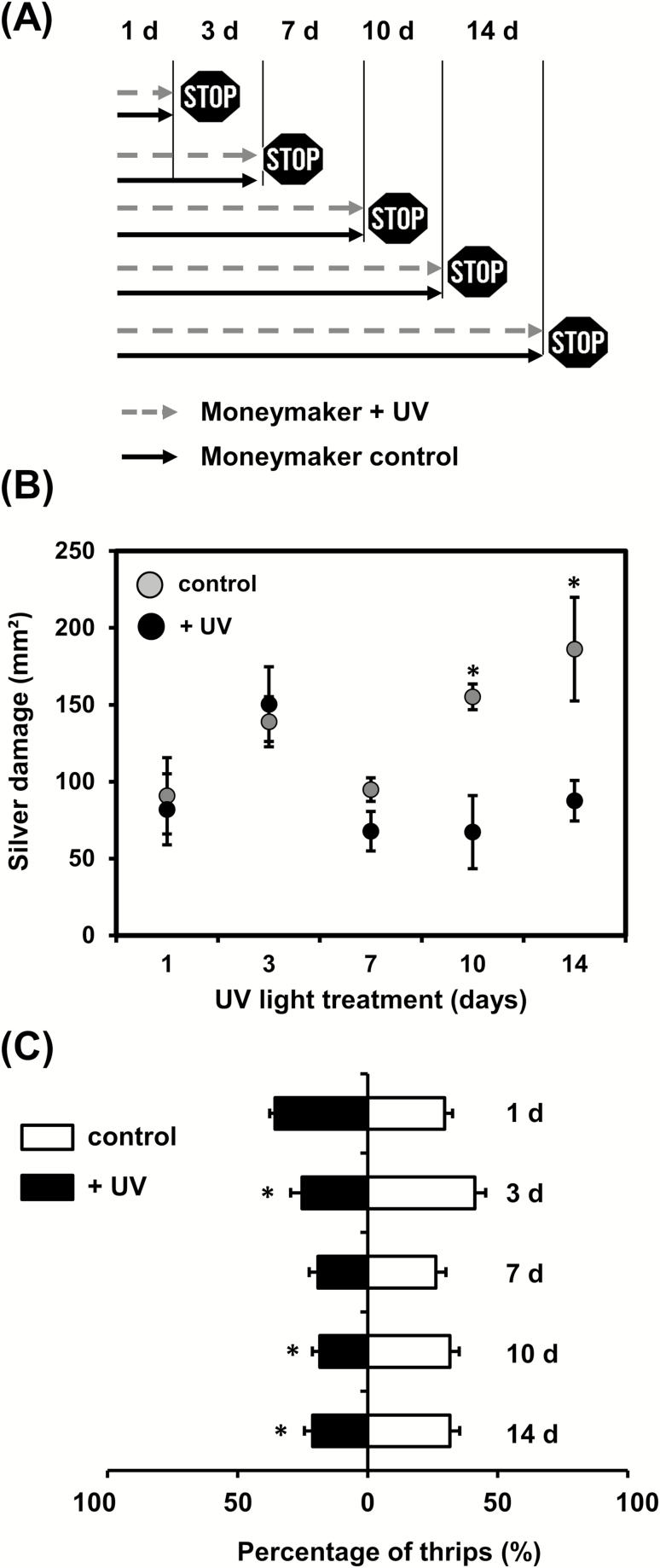
(A) Schematic representation of the time-course experiments to examine responses of tomato ‘Moneymaker’ plants to supplemental UV light. Plants were exposed to 30 min d^−1^ of supplemental UV and tested for resistance to thrips at 1–14 d after the beginning of treatment. (B) Silver damage symptoms determined in plants subjected to non-choice whole-plant thrips bioassays at different time-points. Plant damage was evaluated at 7 d after thrips infestation. Data are means (±SEM) of *n*=5 individual plants and were analysed by Student’s *t*-test: *P≤0.05. (C) Preference of thrips tested by two-choice leaf-disc bioassays at different time-points after the beginning of treatment. Data are the percentage of thrips (+SEM, *n*=5 individual plants) settled on leaf discs taken from control and UV-treated plants. Averaged preference data recorded at 0.5, 1, 2, 3, and 4 h after thrips release are shown. Data were analysed by paired *t*-tests: *P≤0.05.

To determine the dynamics of the UV-mediated induction of resistance to thrips, a time-course experiment was performed (Fig. 2A). Exposure to 30 min d^−1^ of UV significantly decreased silver damage symptoms in plants infested with thrips at 10 d and 14 d after the beginning of the treatment when compared to controls (*P*≤0.05) (Fig. 2B). No significant differences were observed when thrips infestation was performed after shorter periods of treatment.

To assess whether UV affected thrips preference, dual-choice leaf-disc bioassays were carried out for the time-course experiment (Fig. 2C). Thrips preference for leaf discs taken from control or UV-treated plants did not differ at 1 d and 7 d after the beginning of the treatment; however, fewer thrips settled on leaf discs taken from treated plants than on the controls at 3, 10, and 14 d (*P*<0.05).

### UV does not affect the metabolomic profile of leaves or the density of trichomes and their associated volatiles

**Fig. 3.**
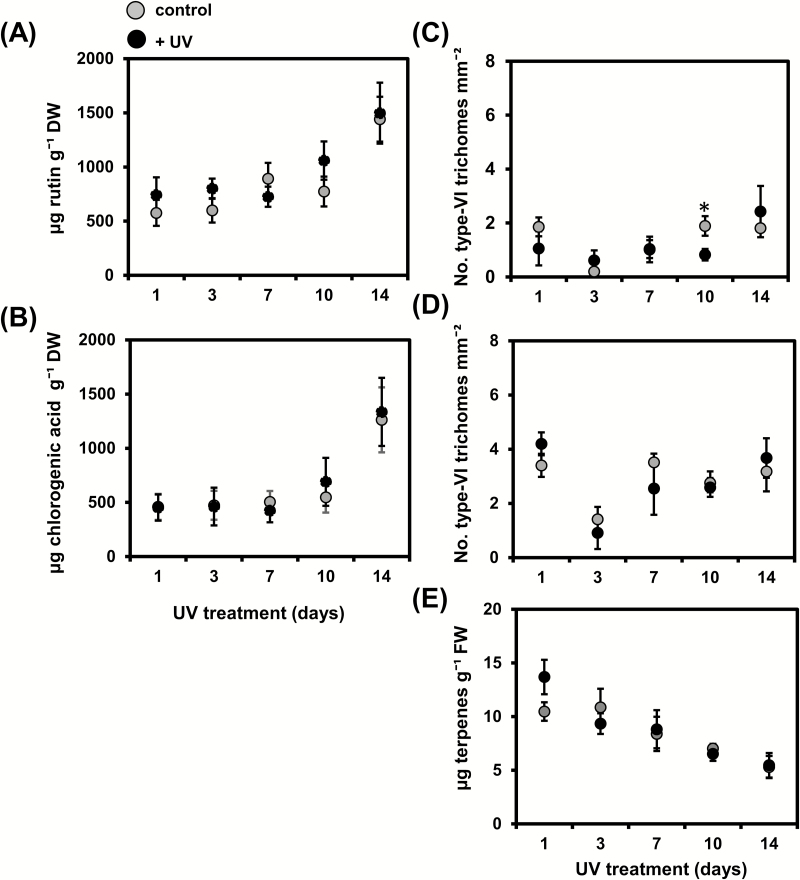
(A) Rutin and (B) chlorogenic acid levels (mean±SEM, *n*=6–10 individual plants) in control and UV-treated tomato ‘Moneymaker’ plants determined at 1–14 d after the start of the treatment. Pooled data from two independent experiments is shown. Type-VI glandular trichome density (mean±SEM, *n*=5 individual plants) on (C) adaxial and (D) abaxial surfaces of leaflets collected from the third/fourth youngest leaf of control and UV-treated plants determined at 1–14 d after the start of the treatment. (E) Total terpene content (mean±SEM, *n*=3–5 individual plants) in trichome-derived leaf exudates of control and UV-treated plants obtained from the third/fourth youngest leaf determined at 1–14 d after the start of the treatment. Data were analysed by Student’s *t*-test: *P≤0.05.

UV did not significantly affect the leaf contents of rutin or chlorogenic acid at any of the points in the time-course experiment (Fig. 3A, B).

The density of type-VI trichomes on the adaxial surfaces of leaves only differed between control and UV-treated plants at 10 d after the beginning of the treatment, with lower density being observed in treated plants (*P*<0.05) (Fig. 3C). No significant differences in trichome density were observed for the abaxial leaf surfaces (Fig. 3D). The total terpene content detected in trichome-derived leaf exudates of UV-treated plants also did not differ from the control (Fig. 3E).

We conducted a non-targeted metabolomic analysis by NMR on plants at 14 d after the beginning of the treatment. A total of 246 signals were detected by ^1^H NMR in the whole-leaf extracts of control and UV-treated plants. Principal component analysis of the detected signals resulted in a model with four principal components explaining 85% of the variance (model statistics: *R*^*2*^*X*=0.85 and *Q*^*2*^=0.474), and indicated no significant separation between the metabolomic profiles of the control and UV-treated plants (Supplementary Fig. S2).

### UV-mediated enhancement of plant resistance to thrips depends on irradiance intensity

**Fig. 4.**
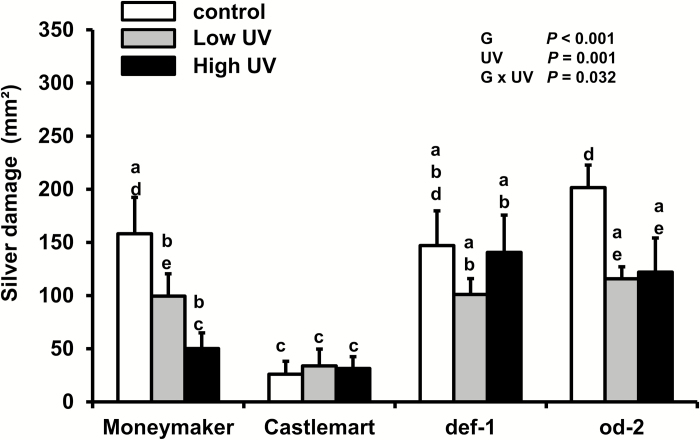
Thrips-associated damage (‘silver damage’ symptoms) for different tomato genotypes in response to UV treatment. Damage was evaluated in ‘Moneymaker’, ‘Castlemart’, *defenceless-1* (*def-1*) and *odorless-2* (*od-2*) plants that were subjected to control, low-, or high-UV irradiance treatments (applied for 30 min d^−1^) for 28 d and subsequently used for non-choice whole-plant thrips bioassays. Silver damage was evaluated at 7 d after thrips infestation. Data are means (+SEM) of *n*=5 individual plants. Different letters indicate significant differences among groups as determined by GLM followed by Fisher’s LSD test (*P*≤0.05). The overall effects of genotype (G), UV, and their interaction are indicated.

In the time-course experiments we observed increases in plant resistance to thrips only at 10 d and 14 d, and therefore we investigated whether resistance was affected by the different intensities of UV irradiance received by the plants at early and late time points as a result of them growing towards the UV source. Two different UV intensities but with the same exposure time (30 min d^−1^) were applied to the *def-1* and *od-2* mutants, their wild-type Castlemart, and to Moneymaker. Under control conditions, *od-2* and *def-1* showed higher susceptibility to thrips than the wild-type Castlemart (Fig. 4). Moneymaker also showed higher susceptibility to thrips when compared to Castlemart. However, under low- or high-UV conditions, significant reductions in silver damage symptoms were observed in Moneymaker and *od-2*, but not in Castlemart or *def-1*. The effects of high-UV irradiance on resistance to thrips were confirmed in a replicated experiment (Supplementary Fig. S3).

### The effects of high-UV irradiance on levels of OPDA, JA-Ile, and SA differ between tomato genotypes

**Fig. 5.**
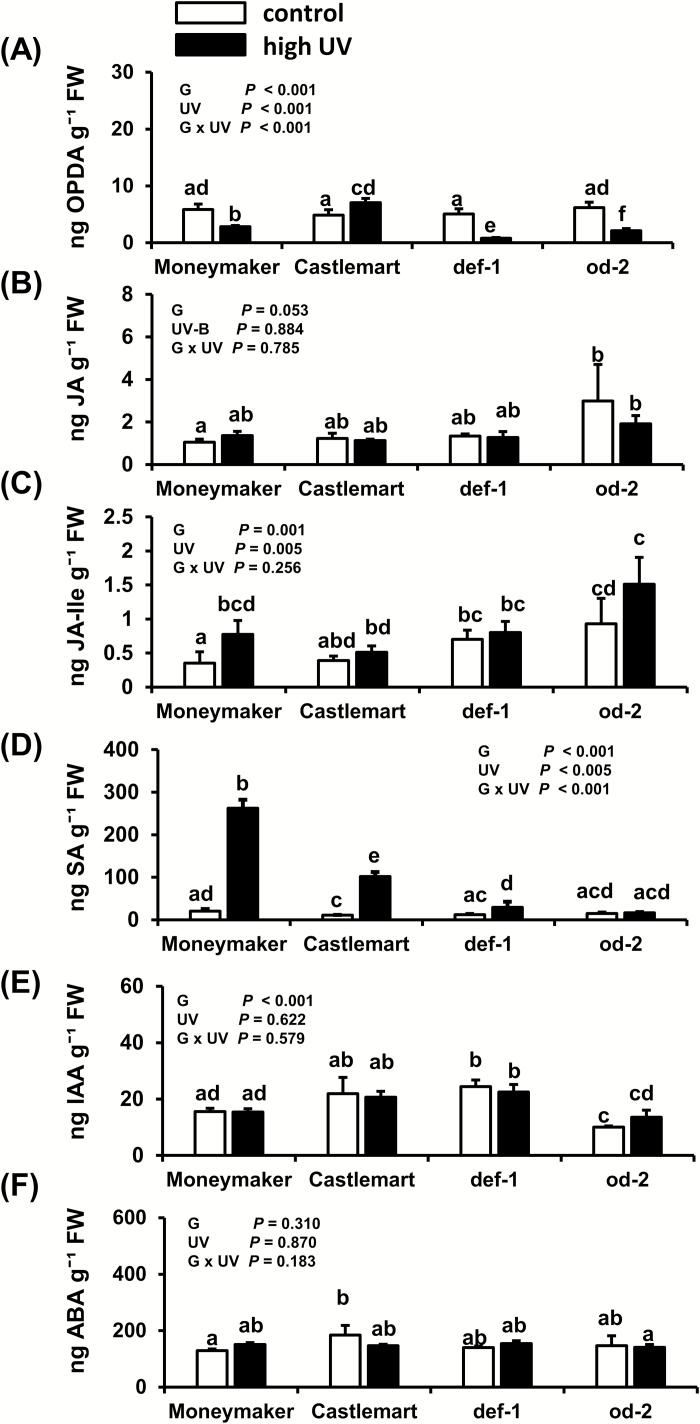
Effects of UV on concentrations of (A) 12-oxo-phytodienoic acid (OPDA), (B) jasmonic acid (JA), (C) jasmonic acid-isoleucine (JA-Ile), (D) salicylic acid (SA), (E) indole-3-acetic acid (IAA), and (F) abscisic acid (ABA) as determined in control and high UV-treated (applied for 30 min d^−1^) tomato genotypes ‘Moneymaker’, Castlemart, *defenceless-1* (*def-1*), and *odorless-2* (*od-2*) at 28 d after the beginning of treatment. The analysis was performed on leaflets collected from the third/fourth youngest leaf. Data are means (+SEM) of *n*=3–5 individual plants and different letters indicate significant differences among groups as determined by GLM followed by Fisher’s LSD test (*P*≤0.05). The overall effects of genotype (G), UV, and their interaction are indicated on each graph.

High-UV significantly decreased OPDA levels in Moneymaker, *def-1*, and *od-2* plants when compared with controls 28 d after the beginning of treatment (Fig. 5A); however, the opposite effect was observed for Castlemart, with a small but significant increase being detected. Levels of JA were not affected by UV in any of the genotypes (Fig. 5B), but the JA-Ile concentration was significantly increased in Moneymaker plants treated with high UV (Fig. 5C). High-UV conditions significantly increased SA concentrations in Moneymaker, Castlemart, and *def-1* plants, but not in *od-2* (Fig. 5D).

UV did not significantly affect IAA and ABA levels when compared between control and treated plants (Fig. 5E, F), but lower IAA levels were observed in *od-2* when compared to the wild-type Castlemart.

### High-UV irradiance induces the expression of JA-responsive genes in Moneymaker and *od-2,* but not in Castlemart and *def-1*

**Fig. 6.**
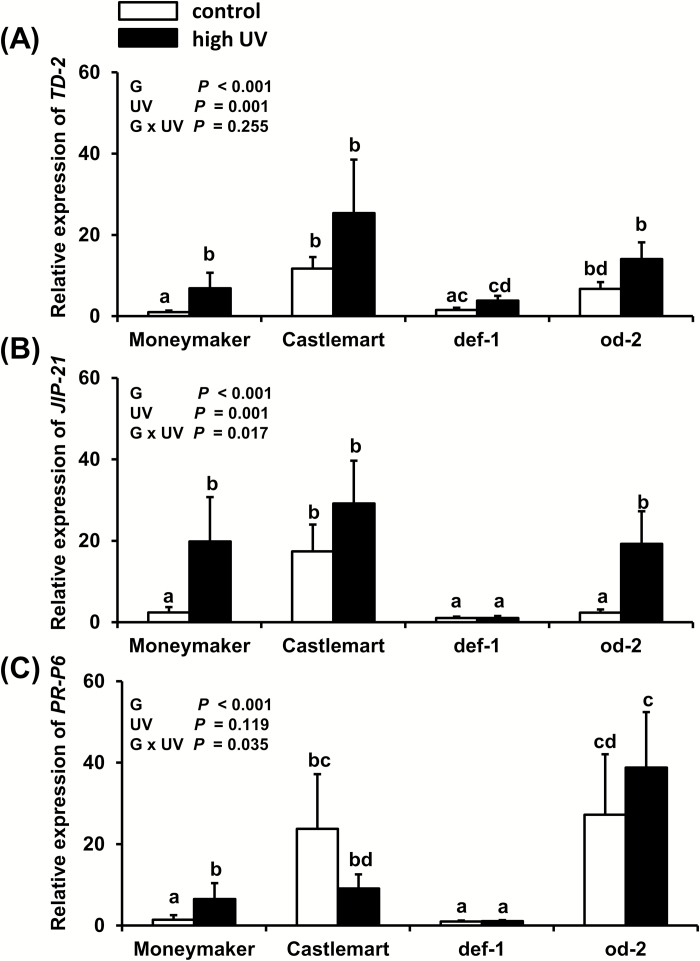
Effects of UV on relative transcript levels of the JA-responsive genes (A) *threonine deaminase-2* (*TD-2*) and (B) *jasmonate inducible protein-21* (*JIP-21*), and (C) the SA-responsive gene the *pathogenesis related-protein 6* (*PR-P6*) as measured in control (no UV) and high UV-treated tomato genotypes ‘Moneymaker’, ‘Castlemart’, *defenceless-1* (*def-1*), and *odorless* (*od-2*) at 28 d after the beginning of treatment (applied for 30 min d^−1^). The analysis was performed on leaflets collected from the third/fourth youngest leaf. Data are means (+ SEM) of relative expression of each treatment group (*n*=5 individual plants, two technical replicates). Different letters indicate significant differences among groups as determined by GLM followed by Fisher’s LSD test (*P*≤0.05). The overall effects of genotype (G), UV, and their interaction are indicated on each graph.

*TD-2* and *JIP-21* expression were significantly increased in high UV-treated Moneymaker and *od-2* plants at 28 d after the beginning of treatment (Fig. 6A, B). In contrast, no significant differences were observed in Castlemart and *def-1*. *PR-P6* had significantly increased expression in Moneymaker plants treated with high-UV, but no significant differences were detected in the other genotypes (Fig. 6C).

### Low- and high-UV irradiance have positive effects on leaf production and stem length, but do not affect type-VI trichome density

**Fig. 7.**
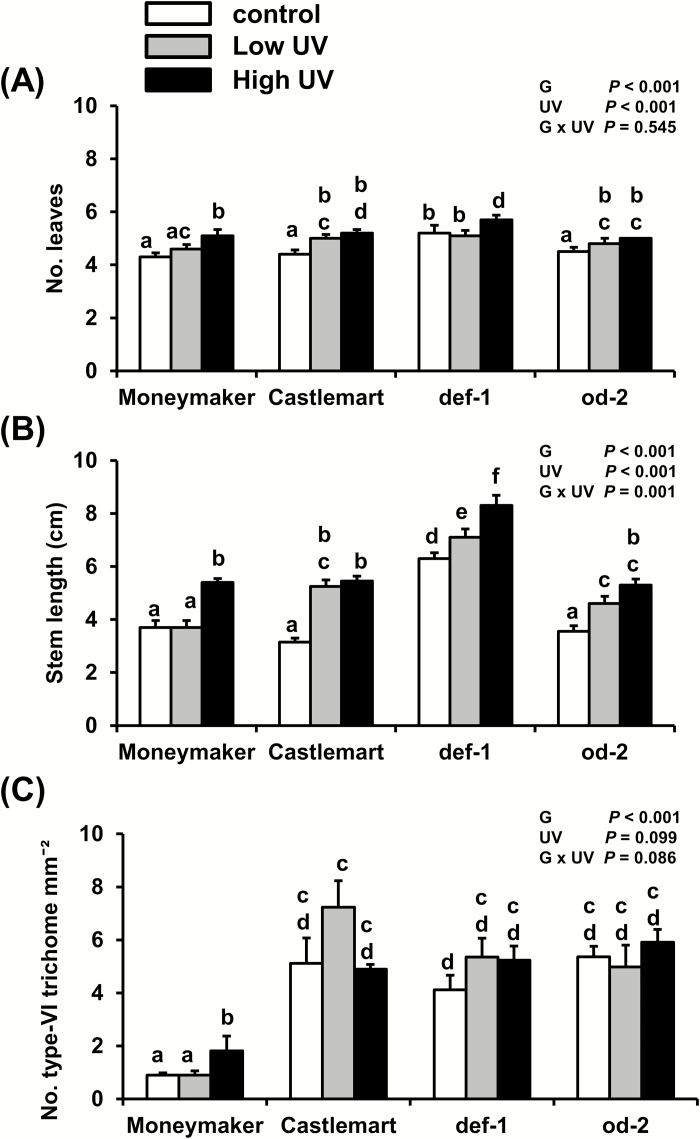
Effects of low- and high-UV irradiance on (A) number of leaves, (B) stem length, and (C) density of type-VI glandular trichomes in the tomato genotypes ‘Moneymaker’, ‘Castlemart’, *defenceless-1* (*def-1*), and *odorless-2* (*od-2*) at 28 d after the beginning of treatment (applied for 30 min d^−1^). Data are means (+SEM) of *n*=5 individual plants and different letters indicate significant differences among groups as determined by GLM followed by Fisher’s LSD test (*P*≤0.05). The overall effects of genotype (G), UV, and their interaction are indicated on each graph.

High-UV irradiance intensity increased the number of leaves in all the genotypes, but low-UV only increased the number in Castlemart and *od-2* (Fig. 7A). Both low- and high-UV irradiance increased the stem length in all four genotypes, with the exception of Moneymaker plants where no effect was seen for low-UV (Fig. 7B). High-UV significantly increased the density of type-VI trichomes on the adaxial leaf surface of Moneymaker plants, but no other effects of UV were observed within the genotypes (Fig. 7C). Moneymaker leaves had significantly lower trichome densities when compared to Castlemart, *def-1*, and *od-2*.

## Discussion

In this study, we demonstrate that UV-mediated enhancement of tomato resistance to *F. occidentalis* depends on the UV exposure time and irradiance intensity, and that the induction of resistance is not explained by UV-associated changes in the leaf metabolome or trichome-mediated defenses, but it might be explained by reinforcement of JA-associated defenses.

We first determined that exposure to 30 min d^−1^ of supplemental UV had the strongest positive effect on plant resistance to thrips, and this effect decreased with longer exposure times (Fig. 1). These results suggest the existence of specific defense-related photomorphogenic responses to different UV exposure times. Numerous studies have described the beneficial effects of solar or supplemental UV-B on plant resistance to herbivorous arthropods (Caputo *et al.*, 2006; Foggo *et al.*, 2007; Mewis *et al.*, 2012; Đinh *et al*., 2013; Mazza *et al.*, 2013; Zavala *et al.*, 2015; Dillon *et al.*, 2018; Qi *et al.*, 2018), including thrips (Mazza *et al.*, 1999; Demkura *et al.*, 2010). However, to the best of our knowledge, there have been no previous reports that show how different UV exposure times affect plant defenses against herbivory. Plant chemical responses to different UV-B exposure times and intensities have been described; for example, de la Rosa *et al.* (2001) reported modifications in the induction of phenolic compounds in seedlings of silver birch (*Betula pendula*) when exposed to different supplemental UV-B irradiation times (ranging from 36–300 min d^−1^).

Our results from time-course experiments also showed that the effects of supplemental UV (30 min d^−1^) on plant defenses against thrips were evident only at 10 d and 14 d after the beginning of the treatment (Fig. 2). Our targeted metabolite analyses of leaves and trichome-related tissues revealed no significant changes after UV exposure at these time points (Fig. 3). This was surprising, as one of the most described UV-mediated changes in plant metabolism is an increased accumulation of phenolic acids and flavonoids, such as quercetin derivatives (de la Rosa *et al.*, 2001; Hectors *et al.*, 2014; Barnes *et al.*, 2016), which is explained by their role in plant photoprotection (Agati *et al.*, 2013). It should be noted that although both UV-B and -A may contribute to the accumulation of UV-absorbing compounds, UV-B plays a major role in the accumulation of flavonoids and phenolic acids (Morales *et al.*, 2010, 2011, 2013). In tomato, UV-B induces production of UV-absorbing compounds in the leaves, but these compounds have not been identified (Barnes *et al.*, 2016). Production of quercetine and kaempferol derivatives is increased by solar UV-B in Arabidopsis (Demkura and Ballaré, 2012; Morales *et al.*, 2013) and also in *Nicotiana attenuata*, together with chorogenic acid (Demkura *et al.*, 2010; Đinh *et al*., 2013). Hence we expected that these phenolic compounds would be responsive to UV in tomato as well. Supplemental UV promotes the production of flavonoids within 1–5 d in Arabidopsis, with levels remaining elevated 13 d after the start of the treatment (Hectors *et al.*, 2014), and induction of phenylpropanoid pathway-related genes can be detected 12 h after exposure to solar UV (Morales *et al.*, 2013). Thus, our sampling regime should have been able to detect similar temporal changes in phenolic concentrations of UV-treated tomato leaves. The lack of induction of chlorogenic acid and the quercetin-derivative rutin (Fig. 3A, B), the main phenolic components in tomato leaves (Larbat *et al.*, 2014), might be explained by species-specific responses to UV, or by the differing UV doses or PAR levels used in our experiments (Götz *et al.*, 2010). A decrease in UV-mediated induction of UV-absorbing compounds has been described under low-PAR conditions (Cen and Bornman, 1990; Gotz *et al.*, 2010; Klem *et al.*, 2012). More recently, Coffey *et al.*, (2017) and Coffey and Jansen (2018) have reported an absence of induction of UV-absorbing compounds in Arabidopsis plants grown outdoors under different UV filters. Interestingly, these experiments were performed across different seasons in a temperate climatic area, i.e. characterized by a lack of extreme temperatures and low hours of direct sunshine, resulting in low daily light integrals, as it was also the case under our experimental conditions. In addition, Hectors *et al.* (2007) found no significant changes in UV-absorbing compounds in Arabidopsis plants subjected to UV-B irradiances between 0–0.1 mW cm^−2^. We applied UV doses within that range (i.e. 0–0.02 mW cm^−2^; Supplementary Table S1), which might explain our results. It is also possible that UV-A might have ameliorated the effects of UV-B exposure (Wilson *et al.*, 2001). Our non-targeted metabolomics analysis by NMR further demonstrated that supplemental UV did not significantly alter the leaf chemical profile of plants at 14 d after the beginning of treatment (Supplementary Fig. S2). However, UV-B might have affected the abundance of other compounds, not included in our targeted chemical analysis, at earlier time points. It was also notable that the density of type-VI trichomes and production of the main volatile allelochemicals associated with them, terpenes, were not affected by supplemental UV (Fig. 3C, D, E). Taken together, these results suggest that UV-mediated induction of tomato defenses against thrips is not associated with changes in constitutive (pre-herbivory) levels of leaf-associated phenolics or with trichome-related traits.

The fact that the UV-mediated enhancement of tomato defenses against thrips was only evident at late time points (10 d and 14 d, Fig. 2) might be explained either by a required minimum number of doses (i.e. days) to elicit the responses or by the different UV irradiance intensities to which the plants were exposed as they grew towards the light source. This prompted us to test whether two UV irradiance intensities with the same daily exposure times could trigger different defense responses. And indeed, our results showed that under high-UV conditions Moneymaker plants experienced a greater reduction in silver damage symptoms than under low-UV (Fig. 4). In a wider comparison of different tomato genotypes, Moneymaker and the trichome-defective mutant *od-2* both displayed increased resistance to thrips under high- and low-UV, whilst the jasmonate deficient mutant *def-1* and the wild-type of the mutants, Castlemart, did not. This experimental set-up tested whether the effect of UV on the plants responses was mediated by the activation of JA defenses or the presence/absence of functional type-VI leaf trichomes. It was notable that Castlemart plants displayed higher thrips resistance levels when compared to both *def-1* and *od-2*, and to Moneymaker. These results confirmed previous reports on the role of JA defenses against thrips in tomato (Li *et al.*, 2002; Escobar-Bravo *et al.*, 2017*a*), and suggest possible diminished constitutive and/or inducible defenses against thrips in Moneymaker.

We further found that reductions in silver damage symptoms in high UV-treated Moneymaker and *od-2* plants was accompanied by an overall increase in JA-Ile levels (Fig. 5C), as well as by induction of JA-responsive genes (Fig. 6A,B). UV-B has been reported to increase constitutive levels of JA-Ile in Arabidopsis, tobacco (*Nicotiana tabaccum*), rice (*Oriza sativa*), and maize (*Zea mays*), and also to increase the levels of JA-Ile and defense-related secondary metabolites after simulated herbivory in these species, resulting in enhanced resistance to lepidopteran insects (Qi *et al.*, 2018). It is known that the JA signaling pathway plays a fundamental role in tomato defenses against thrips (Li *et al.*, 2002; Escobar-Bravo *et al.*, 2017*a*) and therefore UV-mediated reinforcement of these defenses might explain the increased resistance that we observed. In particular, increased activity of proteinase inhibitors, which are JA-responsive defense proteins involved in plant resistance to arthropod herbivores (Farmer *et al.*, 1992), might have negatively affected thrips feeding and performance (Outchkourov *et al.*, 2004). UV-mediated increases in jasmonate levels might also have strengthen inducible defense responses in tomato after thrips infestation, i.e. through priming mechanisms (Martinez-Medina *et al.*, 2016), but this needs further investigation.

Our analyses also showed increased levels of SA under high-UV conditions in Moneymaker, Castlemart, and *def-1* plants, but not in *od-2* (Fig. 5D);. however, expression of the SA-responsive gene *PR-P6* was only significantly induced in Moneymaker (Fig. 6C). Induction of SA and pathogen-related (PR) proteins have been reported in response to increasing UV-B exposure times in Arabidopsis (Surplus *et al.*, 1998; Mackerness *et al*., 1999), and UV-B induces SA accumulation in barley (Bandurska and Cieślak, 2013), wheat (Kovács *et al.*, 2014), and tobacco leaves (Fujibe *et al.*, 2000). The increases in SA and PR-proteins in Arabidopsis have been explained by the generation of ROS (Surplus *et al.*, 1998; Mackerness *et al.*, 2001), but whether a similar explanation might apply to tomato under our experimental conditions is unknown. Furthermore, we did not test whether the increased SA levels were a UV-associated stress response accompanied by increases in UV-absorbing compounds. In Arabidopsis under natural sunlight, UV-associated photomorphogenic responses controlled by the UVR8 receptor share many similarities with those related to defense and stress-related signaling, including accumulation of JA and induction of SA-related genes (Morales *et al.*, 2013). Interestingly, induction of SA signaling has been associated with increased plant susceptibility to thrips, due to its antagonistic effect on JA-induced defenses (Leon-Reyes *et al.*, 2009; Abe *et al.*, 2012). In our study, however, UV-mediated induction of both the JA- and SA-signaling pathways resulted in increased tomato resistance to thrips. This absence of JA–SA antagonistic effects might be explained by the relative plant levels of both hormones, as different JA:SA ratios can result in neutral, antagonistic, or synergistic effects (Mur *et al.*, 2006). Whether the induction of SA might alter tomato resistance against biotrophic pathogens that are susceptible to the activation of this signaling pathway (Pieterse *et al.*, 2012) needs further research.

The phytohormones auxin and ABA can modulate the SA–JA backbone of the plant immunity signaling network (Pieterse *et al.*, 2012), and their concentrations might be affected by UV. Both increased ABA and reduced auxin accumulation have been observed to occur upon exposure to moderate to high UV-B levels in different species, mostly associated with UV stress-related responses (Hayes *et al.*, 2014; Vanhaelewyn *et al.*, 2016). Here, we found that ABA and auxin levels were not altered by high-UV conditions at 28 d after the beginning of the treatment (Fig. 5E, F). This suggests that our UV conditions did not induce stress-related responses in tomato plants, and that UV-mediated enhancement of tomato resistance to thrips might not be mediated by changes in the constitutive levels of these hormones. However, UV significantly affected some plant growth parameters, namely the number of leaves and stem length (Fig. 7A, B). Auxins regulate plant growth and development, including stem and petiole elongation (Deng *et al.*, 2012; Naseem *et al.*, 2015). Fluctuations in auxin levels prior to our sampling time might explain the increased stem length in UV-treated plants.

Moneymaker and the *od-2* mutant experienced responses to UV that were not observed in Castlemart, such as increased resistance to thrips (Fig. 4) and enhanced expression of JA-responsive genes (Fig. 6A, B). Deficiencies in different aspects of trichome-related traits might have potentiated their sensitivity/responses to UV. Plant leaf hairs can protect underlying photosynthetic tissues against UV radiation by reflecting both it and longer-wavelength radiation, and their absence or removal increases plant sensitivity to UV (Karabourniotis *et al.*, 1992, 1993, 1995; Holmes and Keiller, 2002; Manetas, 2003; Yan *et al.*, 2012). The density of type-VI trichomes was lower in Moneymaker than in Castlemart (Fig. 7C), which might result in reduced production of trichome-associated allelochemicals, which are positively correlated with trichome density (Escobar-Bravo *et al.*, 2017*a*). In *od-2*, the trichome density was not reduced in comparison to the Castlemart wild-type, but a strong reduction in the type-VI trichome-derived allelochemicals terpenes and flavonoids has been reported for this genotype (Kang *et al.*, 2010*a*). Although the specific role of type-VI trichome-derived flavonoids in tomato photoprotection has not been studied, a reduced accumulation of this compound in Moneymaker and *od-2* might have increased the penetration of UV radiation into the underlying plant tissues.

Taken together, our results show that both short daily exposure times and specific intensities of UV radiation can increase tomato resistance to thrips herbivory, probably via the reinforcement of JA defenses. They might also counterbalance the reported negative effects of UV-B on plant growth that produce dwarf phenotypes (Ballaré *et al.*, 2011), although this requires additional research. Our results have important implications for agriculture, as the use of supplemental UV radiation can enhance crop protection against pests and thus might reduce the need for pesticides.

## Supplementary data

Supplementary data are available at *JXB* online.

Fig. S1. Spectral irradiance of the UV lamps used in the experiments.

Fig. S2. Principal component analysis of the metabolic responses of control and UV-treated Moneymaker tomato plants at 14 d after the start of treatment.

Fig. S3. Effects of high-UV irradiance on the resistance to thrips of different tomato genotypes.

Table S1. Measurements of UV irradiance at the plant canopy level.

Table S2. Transitions or specific pairs of *m*/*z* values associated with the precursors and fragment ions of the analytes measured by LC/MS.

Table S3. List of primers used for qRT-PCR analysis.

Supplementary Figures S1-S3 and Table S1-S3

## Data deposition

The raw data for Figs 1–7 and Supplementary Figs S2 and S3 are available at Dryad Data Repository: http://dx.doi.org/10.5061/dryad.55n17h8 (Escobar-Bravo *et al.*, 2019).

## Abbreviations

ABA: abscisic acid
IAA: indole-3-acetic acid
JA: jasmonic acid
JIP-21: *jasmonate inducible protein-21*
OPDA: 12-oxo-phytodienoic acid
PR-P6: *pathogenesis related-protein 6*
TD-2: *threonine deaminase*
SA: salicylic acid
