# Supplementary material for: Ultraviolet radiation exposure time and intensity modulate tomato resistance to herbivory through activation of jasmonic acid signaling
Source: J Exp Bot. 2018 Oct 9;70(1):315–27. doi: 10.1093/jxb/ery347 (PMC6305188; doi:10.1093/jxb/ery347)
Supplement: Supplementary Figures S1-S3 and Table S1-S3 [file ery347_suppl_supplementary_figures_s1-s3_table_s1-s3.pdf]

Supplementary data

Ultraviolet radiation exposure time and intensity modulate tomato resistance to herbivory through activation of jasmonic acid signaling

Rocío Escobar-Bravo[1], Gang Chen[1], Hye Kyong Kim[1], Katharina Grosser[2,3], Nicole M. van Dam[2,3], Kirsten A. Leiss[1*], Peter GL. Klinkhamer[1].

[1]Plant Science and Natural Products, Institute of Biology Leiden (IBL), Leiden University. Sylviusweg 72, 2333BE Leiden (The Netherlands).

[2]Molecular Interaction Ecology, German Center for Integrative Biodiversity Research (iDiv), Halle-Gena-Leipzig, Deutscher Platz 5e, 04103 Leipzig (Germany).

[3]Friedrich Schiller University Jena, Institute of Biodiversity, Dornburger-Str. 159, 07743 Jena (Germany).

*Current address: Wageningen University & Research, Business Unit Horticulture, Violierenweg 1, 2665MV Bleiswijk (The Netherlands).

To whom correspondence should be addressed:

Rocío Escobar-Bravo, E-mail: r.bravo@biology.leidenuniv.nl

This file contains information on supplementary Fig. S1, S2 and S3, Table S1, S2 and S3.

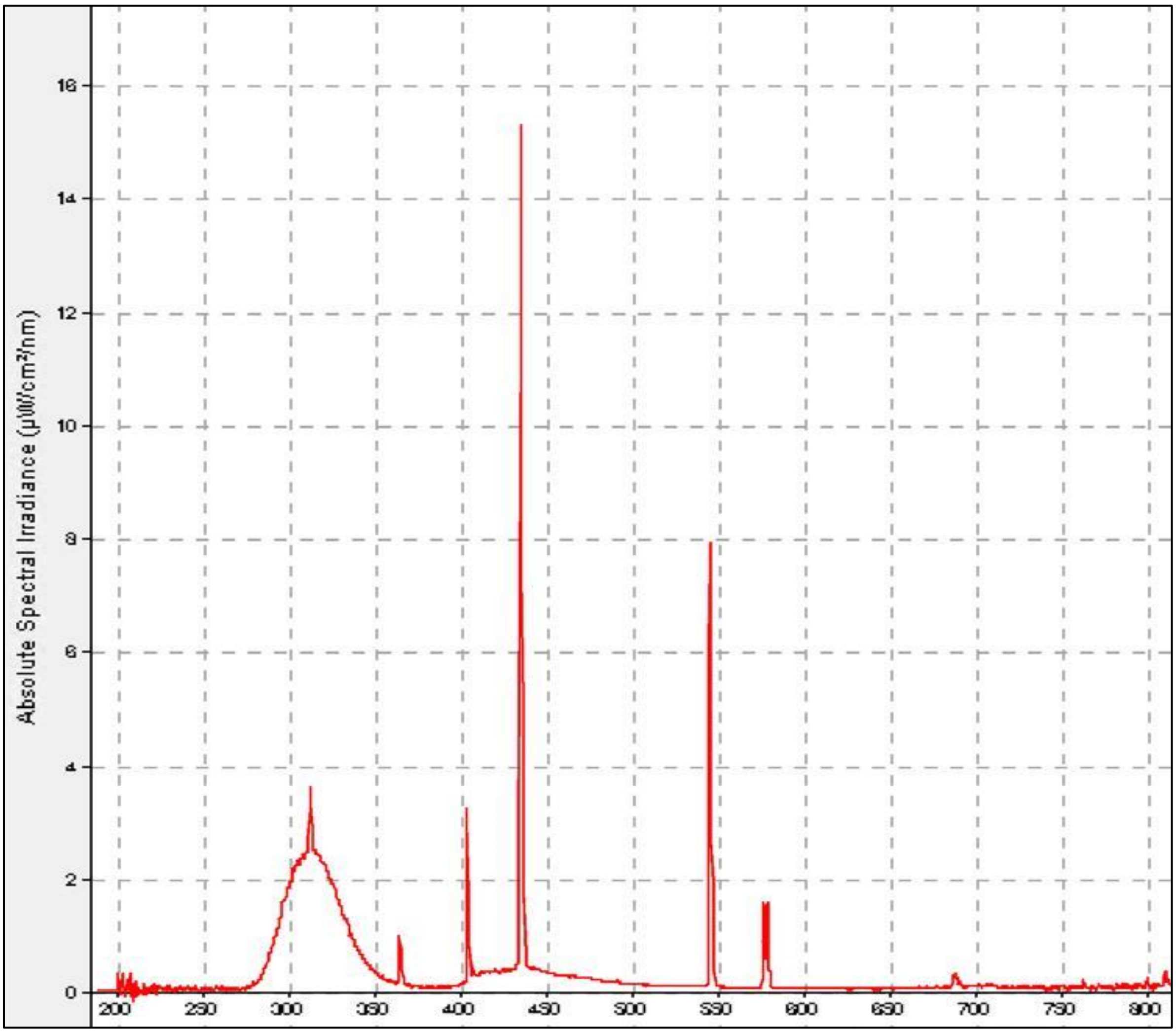

Figure S1. Spectral irradiance of the UV lamps after wrapping with white cellulose filters. UV lamps emit residual (< 10% of total PAR) blue (λ 400-450 nm), green (λ 520-560), and yellow (λ 560-590 nm) light. PAR levels in the UV growth chamber did not differ from the control (no UV) growth chamber.

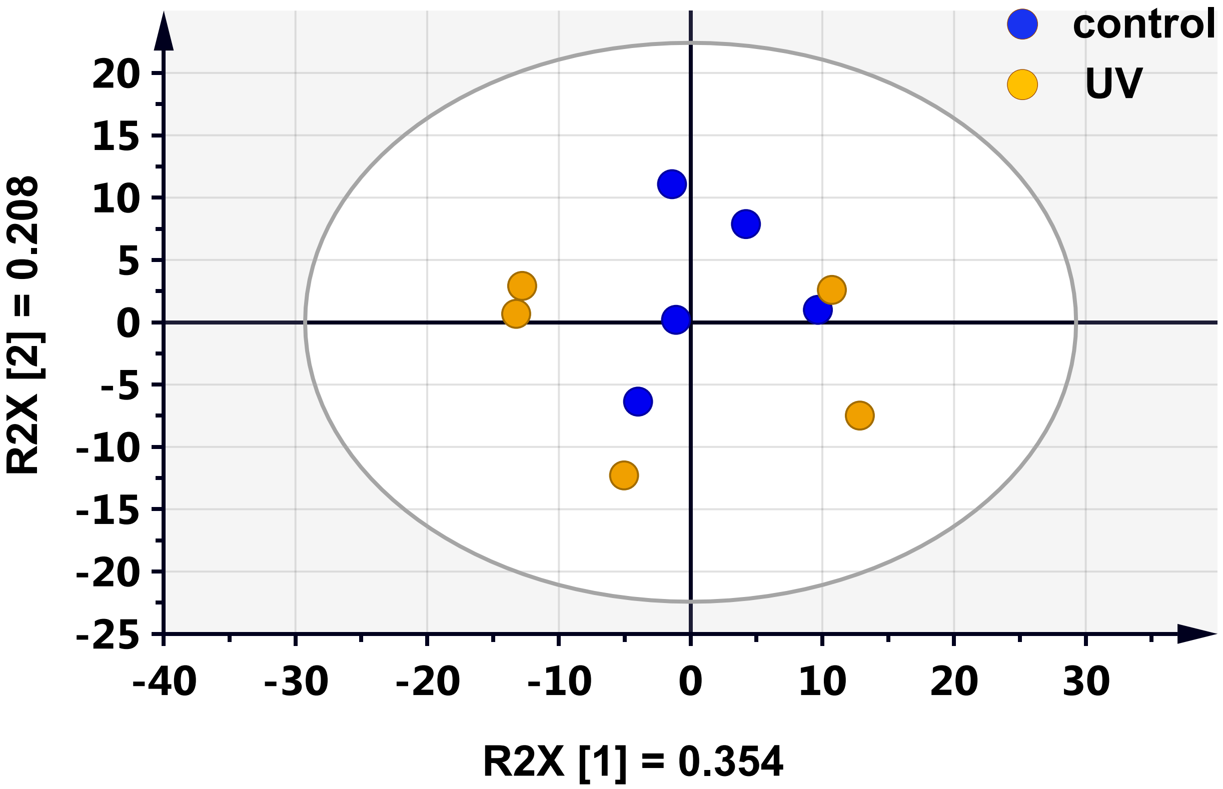

Figure S2. Metabolic responses in control and UV-treated Moneymaker tomato plants was determined at 14 days after the start of the light treatments. Leaf metabolites were analyzed by NMR on leaflets collected from the third/fourth youngest leaf. The ^{1}H NMR spectra was subjected to principal component analysis and resulted in a model with four principal components explaining 85% of the variance. The first two components, explaining 35.5 and 20.8% of the variance, are represented in the graph. The ellipse defines the Hotelling's T2 confidence region (95%).

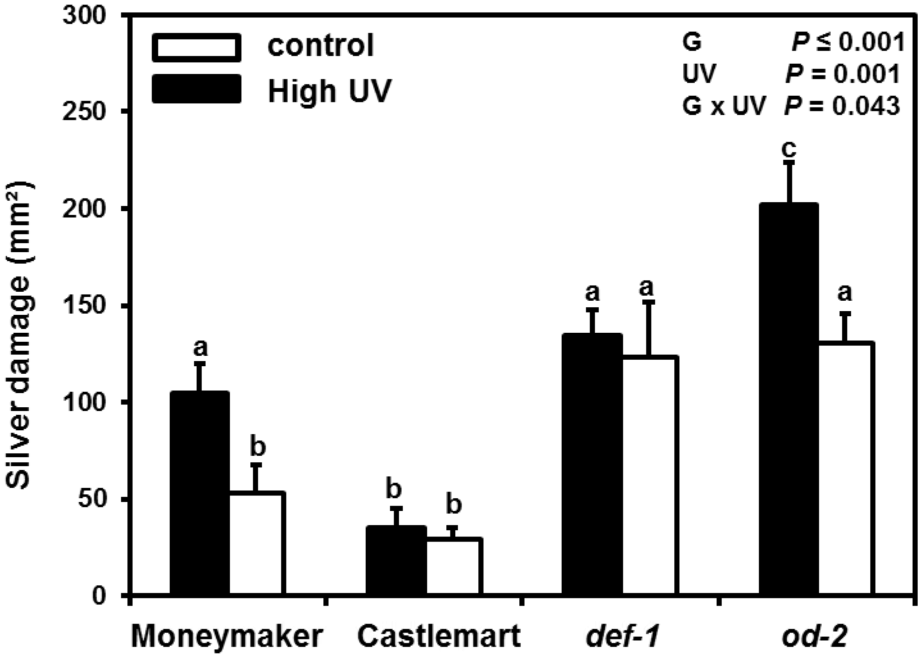

Figure S3. Thrips-associated damage (i.e. 'silver damage' symptoms) was evaluated in Moneymaker, Castlemart, *def-1* and *odorless-2* (*od-2*) plants that were first subjected to control or high UV irradiance treatments for 28 days and subsequently used for non-choice whole-plant thrips bioassays. Silver damage was evaluated at 7 days after thrips infestation. Supplemental control (no UV) or high UV irradiance conditions were applied for 30 min d^{-1}. Values represent the mean + SEM (n = 7-9 individual plants). Different letters above bars denote significant differences among groups tested by GLM followed by Fisher´s LSD test ($P \leq 0.05$).

Table S1. Measurements of UV irradiance were carried out at the plant canopy level at day 1, 3, 7, 10 and 14 after the start of the light treatments. The integrated irradiance (μW cm^2) was converted to kJ m^2 d^{-1} to estimate the daily UV doses.

	Integrated Irradiance (μW cm^{-2})			Total daily UV doses (kJ m^2 d^{-1})					
**Measurements**	**UV-B 280-315 nm**	**UV-A 315-400 nm**	**UV 280-400 nm**	**15 min**	**30 min**	**45 min**	**60 min**	**120 min**	**180 min**
**Day 1**	**7.6**	**9.25**	**16.85**	0.15	0.30	0.454	0.606	1.212	1.818
**Day 3**	**7.6**	**9.06**	**16.66**	0.149	0.299	0.449	0.599	1.198	1.797
**Day 7**	**8.77**	**10.2**	**19.01**	0.175	0.342	0.513	0.684	1.36	2.04
**Day 10**	**9.19**	**10.76**	**19.95**	0.175	0.359	0.538	0.718	1.436	2.154
**Day 14**	**10.89**	**12.6**	**23.49**	0.211	0.422	0.634	0.845	1.6	2.4
**Averaged**	**8.81**	**10.37**	**19.19**	**0.172**	**0.344**	**0.517**	**0.69**	**1.360**	**2.04**

Table S2. Transitions or specific pair of m/z values associated to the precursors and fragment ions of the analytes measured by LC/MS.

Analyte	Q1 [m/z] → Q3 [m/z][a]	CE [V]	Standard
ABA	(-)263.13 → 153.00	9	D6-ABA
JA	(-)209.12 → 59.00	12	D6-JA
JA-Ile	(-)322.20 → 130.00	19	D6-JA-Ile
SA	(-) 137.02 → 93.00	15	D6-SA
IAA	(+) 176.07 → 130.00	-14	D5-IAA
D6-ABA	(-) 269.17 → 159.00	10	
D6-JA	(-)215.15 → 59.00	10	
D6-JA-Ile	(-) 328.24 → 130.00	19	
D6-SA	(-)141.05 → 97.00	15	
D5-IAA[b]	(+) 181.10 → 135.00	-14	
	(+) 181.10 → 134.00	-14	
	(+) 181.10 → 133.00	-14	
OPDA	(-) 291,00 → 165.00	18	D6-JA-Ile

CE: collision energy

a Resolution: Q1: 0.7, Q3: 22

b Analyzed as the sum of all three transitions

Table S3. Nucleotide sequence of primers used for qRT-PCR analysis.

Target gene	Gene identification	Forward Primer 5' → 3'	Reverse Primer 5' → 3'
PR-P6	Solyc00g174340	GTACTGCATCTTCTTGTTTCC A	TAGATAAGTGCTTGATGTGCC
TD-2	Solyc09g008670	TGCCGTTAAAAATGTCACCA	ACTGGCGATGCCAAAATATC
JIP-21	Solyc03g098790	ACTCGTCCTGTGCTTTGTCC	CCCAAGAGGATTTTCGTTGA
Actin	Solyc03g078400	TTAGCACCTTCCAGCAGATGT	AACAGACAGGACACTCGCACT
